# Income inequalities in stroke incidence and mortality: Trends in stroke-free and stroke-affected life years based on German health insurance data

**DOI:** 10.1371/journal.pone.0227541

**Published:** 2020-01-16

**Authors:** Juliane Tetzlaff, Siegfried Geyer, Fabian Tetzlaff, Jelena Epping

**Affiliations:** 1 Medical Sociology Unit, Hannover Medical School, Hanover, Germany; 2 Institute for General Practice, Hannover Medical School, Hanover, Germany; Sciensano, BELGIUM

## Abstract

**Background:**

Due to substantial improvements in prevention and therapy, stroke incidence and mortality rates have decreased during the last decades, but evidence is still lacking on whether all socioeconomic groups benefited equally and how the length of life affected by stroke developed over time. Our study investigates time trends in stroke-free life years and life years affected by stroke. Special emphasis is given to the question whether trends differ between income groups, leading to decreasing or increasing social inequalities.

**Methods:**

The analyses are based on claims data of a German statutory health insurance company of the two time periods 2006–2008 and 2014–2016. Income inequalities and time trends in incidence and mortality risks were estimated using multistate survival models. Trends in stroke-free life years and life years affected by stroke are analysed separately for income groups by applying multistate life table analyses.

**Results:**

Stroke incidence and mortality risks decreased in men and women in all income groups. While stroke-free lifetime could be gained in men having higher incomes, improvements in mortality counterbalanced decreasing incidences, leading to increases in life years affected by stroke among men of the lower and higher income group. Among women, no significant changes in life years could be observed.

**Conclusions:**

Changes in stroke-affected life years occur among men in all income groups, but are more pronounced in the higher income group. However, irrespective of the income group the proportion of stroke-affected life years remains quite stable over time, pointing towards constant inequalities. Further research is needed on whether impairments due to stroke reduced over time and whether all socioeconomic groups are affected equally.

## Introduction

Despite substantial improvements in prevention and therapy, stroke is still a major public health concern causing serious impairments in the affected individuals. Stroke is one of the leading causes of disabilities, poor quality of life, and long-term care needs [1–3] resulting in higher healthcare utilisation and healthcare expenditures [4–6]. As the risk of stroke incidence is strongly associated with age, rising life expectancy and population aging are expected to foster increasing numbers of strokes and individuals living with its consequences [7, 8]. However, research investigating not only the deployment in incidence over time but the average lifespan affected by stroke is rare and little is known about time trends with respect to social disparities.

If life expectancy increases, the question whether these years gained are spent in good health or an extended lifespan in ill health has to be expected, becomes a crucial public health issue. In this context, stroke is of particular interest as it presents one of the most frequent causes of long-term impairment and disability. According to Fries, improvements in prevention lead to a postponement of disease onset into higher ages over time. Thus, healthy life years are gained and morbidity is compressed into shorter periods towards the end of life [9, 10]. In contrast, Gruenberg put up the hypothesis that medical progress may increase the lengths of survival after disease onsets, thus causing an expansion of life years spent in poor health [11]. A dynamic equilibrium is achieved if the proportion of life years spent in ill health remains constant or if impairments due to diseases become less severe over time [12].

Research investigating time trends in stroke incidence in high-income countries mostly points towards constant [13] or decreasing rates [3, 14, 15]. This applies also to Germany, where the majority of studies reported declining or constant incidence rates over time [16–18]. Due to improvements in treatment and rehabilitation mortality after stroke has declined continuously over the last decades, thus leading to prolonged survival after stroke incidence [3, 19–22]. Because of these mortality declines, stable proportions of lifetime prevalence have been reported for Germany despite decreasing or constant rates of stroke incidence [23]. Due to these interactions between incidence and mortality, it is crucial not to rely on analyses of incidences only, but to include information on mortality as well if the development of the average lifespan spent free of stroke and that affected by stroke is to be examined.

The importance of socio-economic status as predictor for stroke incidence and stroke-related mortality has been emphasised by many studies [23–28]. Previous research shows that health inequalities in stroke exist in low- and middle-income countries as well as in high-income countries [25]. Low socioeconomic status is associated with a higher prevalence of risk factors (e.g. hypertension, smoking, BMI, physical inactivity, diabetes, diet, and alcohol intake) [24, 25, 29], with higher incidence risks, increased stroke severity, and higher stroke mortality [23–28, 30].

However, previous research lacks evidence on how the length of life spent free of stroke and affected by stroke developed over time and whether time trends are socially patterned. The present study is aiming to step into this gap by exploring trends in stroke-free life years and those affected by stroke. This is achieved by combining information on the development of stroke incidence and mortality after stroke incidence as well as in the stroke- free population. Special emphasis is given to the question whether these time trends differ between income groups, leading to narrowing or widening health inequalities over time.

The analyses are based on claims data of a large German statutory health insurance, which makes it possible to perform analyses based on large case numbers and to obtain robust estimates from a single dataset. The study addresses the following three research questions:

- Have stroke incidence risks developed similarly among the different income groups?
- Have mortality risks after stroke incidence and mortality risks without stroke developed similarly among the different income groups?
- Are there income inequalities in the number of life years spent free of stroke and those affected by stroke?
- Are there differing time trends between income groups in life years without stroke and after stroke incidence?

## Materials and methods

### Ethics statement

Our study is based on claims data, i.e., on routinely collected data of a statutory health insurance provider. We confirm that all data are fully anonymised before we accessed them. The use of this sort of data for scientific purposes is regulated by federal law. The data protection officer of the Statutory Local Health Insurance of Lower Saxony (AOK Niedersachsen) has approved its use.

### Data

The analyses are based on claims data of a large German statutory health insurance located in Lower Saxony, the AOK Niedersachsen (AOKN). The data were collected for accounting purpose and cover approximately one third of the population of Lower Saxony. The data contain demographic and socioeconomic information, in- and outpatient diagnoses coded according to the International Classification of Disease 10^th^ revision (ICD-10), and date of death for all individuals deceased over the observation period. With respects to age and sex distributions, the data are representative for the population of Lower Saxony as well as for the German population. However, as lower socioeconomic groups are overrepresented, the insurance population differs from the general population in terms of income level and occupational positions. Individuals having very high incomes and self-employed individuals are underrepresented as they are usually insured by a private health insurance company.” [31]. In Germany, health insurance coverage (private or statutory insurance) is mandatory to all inhabitants. Within the statutory health care system, the dimension of health care coverage is the same for all insured individuals. Claims data of statutory health insurances depict health care activities fairly complete as all payments from insurers to providers are registered. As statutory health insurance is part of the welfare state system, almost 90% of the German population are insured by a statutory health insurance provider [32].

The data are available for the years 2005 to 2016. Single years of observation have been combined into time periods as case numbers of stroke incidence and death after stroke by income group are limited. Therefore, all analyses are based on comparisons of two time periods 2006–2008 and 2014–2016. The analyses are performed for all insured individuals aged 50 years and older.

### Income

In Germany, employers are legally bound to report on gross income of their employees to statutory health insurances. Moreover, the data contain information on pensions payments received from the German Pension Insurance. In this study, income is classified relative to the German average pre-tax income in a given year reported by the Federal Statistical Office. Thus, grouping varies between years in absolute terms but remains constant in relative terms, accounting for increasing income level. Furthermore, income was adjusted for inflation, which enables direct comparability between years as purchasing power is kept constant over time. As multiple transitions have to be analysed and case numbers are restricted within income groups, income was classified in two groups: lower (≤60% of the annual German average income) and higher income (>60% of the average income). In both periods, the proportion of missing information on income amounts to 16%. Since the composition of the group of individuals with missing information on income is very heterogeneous, the interpretation of the results is very difficult. Therefore, all analyses are limited to individuals having information on income.

### Definition of stroke incidence

Cases of stroke incidences are identified on the basis of inpatient diagnoses (ICD-10 I60 to I64). We did not distinguish between different types of strokes as coding accuracy has improved over time, leading to a strong reduction of unspecified strokes and to increasing numbers of cerebral infarctions and haemorrhages. Incidence cases are restricted to individuals having a stroke diagnosis in 2006–2008 or 2014–2016 and who had a stroke-free period of one year preceding their diagnosis.

### Statistical analyses

**Fig 1:**
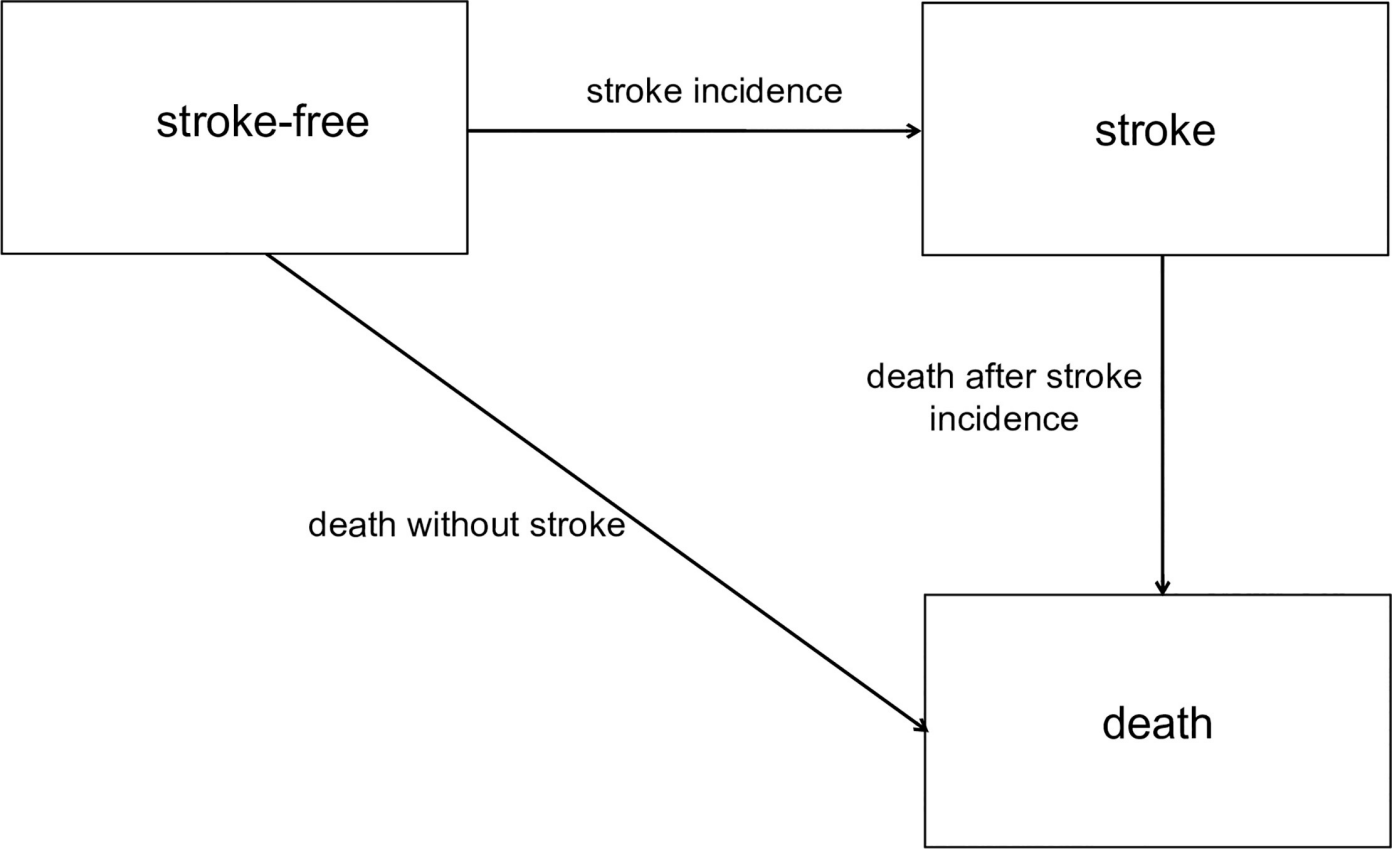
Illness-death model with three transitions: Stroke incidence, death without stroke, and death after stroke incidence.

In this study, three types of events are considered: ***stroke incidence***, ***death without stroke***, and ***death after stroke incidence***. The estimates are based on multistate analyses using an illness-death model without recovery, which is defined by two living states (stroke-free and stroke) and one death state (Fig 1). Between these states, three transitions are possible: 1) stroke-free to stroke (stroke incidence), 2) stroke-free to death (death without stroke), and 3) stroke to death (death after stroke incidence).

Income inequalities and time trends in the risk of these transitions were estimated by fitting proportional hazard survival models with constant baseline hazards defined over time within the two periods. The models used for estimating ***income inequalities*** include the income variable, age, and observation period. The models applied for estimating ***time trends in incidence and mortality*** are stratified for income group and include period and age. The models contain a covariate for age in single-year age groups, which varies with calendar year (centered to the middle of the age interval 50 to 95+ and entered in the model as second-degree polynomial). All models are stratified for the type of event and for sex.

Based on predicted age-specific hazard rates of the three types of transitions, the expected number ***life years free of stroke and affected by stroke*** were estimated using multistate life table analyses. According to the methodological principles of multistate life table analyses, age-specific hazard rates of all three transitions are needed to estimate the expected number of life years in the states considered [33, 34]. These hazard rates were also estimated by fitting proportional hazard models with constant baseline hazard over time and by using the same age variable. The models were estimated separately for each transition and are stratified for observation period and sex. Based on these models, age-specific hazard rates were predicted by using post-estimation commands. These hazard rates were used as input for the multistate life table analyses. Multistate life table calculations are mainly based on matrix multiplication, which was conducted in R 3.5.1 following the methodology described by Palloni [35]. Data management and the estimation of the proportional hazard models are performed using Stata MP 14.2. All confidence intervals were estimated by drawing 1000 bootstrap samples.

## Results

In total, 42’966 incident stroke cases with 5’079’764 person-years of exposure occurred over both observation periods. The number of deaths without stroke amounts to 163’172 with 5’050’342 person-years of exposure. Among the stroke incident individuals, a total of 13’608 deaths and 51’833 person-years were observed. As analyses are restricted to older age groups and include many individuals after retirement age, a substantial proportion of the insured individuals belongs to the lower income group. This holds especially for women, reflecting the overall lower income level compared to men (Table 1).

**Table 1:** Descriptive statistics of the number of insured individuals, exposures in person-years, and number of events by income group, sex and period.

		Men			Women		
Income		Lower	Higher	Total	Lower	Higher	Total
	**Period 1: 2006–2008**						
**Stroke incidence**	*N*	208,785	190,140	398,925	406,475	103,472	509,947
	*Exposures*	551,861	525,793	1,077,654	1,105,139	283,790	1,388,929
	*Events*	5,887	3,537	9,424	10,641	2,035	12,676
**Death without stroke**	*N*	206,410	188,663	395,073	402,471	102,542	505,013
	*Exposures*	547,834	523,316	1,071,150	1,098,804	282,191	1,380,995
	*Events*	23,370	11,574	34,944	38,977	6,717	45,694
**Death after stroke incidence**	*N*	5,883	3,537	9,420	10,639	2,035	12,674
	*Exposures*	7,356	4,470	11,826	11,800	2,362	14,162
	*Events*	1,671	934	2,605	3,891	721	4,612
	**Period 2: 2014–2016**						
**Stroke incidence**	*N*	215,463	222,210	437,673	384,672	139,545	524,217
	*Exposures*	566,013	621,835	1,187,848	1,036,877	388,456	1,425,333
	*Events*	5,756	3,770	9,526	8,704	2,636	11,340
**Death without stroke**	*N*	212,936	220,515	433,451	381,218	138,178	519,396
	*Exposures*	561,832	618,833	1,180,665	1,031,519	386,013	1,417,532
	*Events*	24,483	12,849	37,332	35,401	9,801	45,202
**Death after stroke incidence**	*N*	5,753	3,768	9,521	8,704	2,630	11,334
	*Exposures*	7,492	4,930	12,422	10,053	3,370	13,423
	*Events*	1,609	923	2,532	2,909	950	3,859

### Income inequalities in stroke incidence and mortality risks

Income inequalities in the risk of stroke incidence, death without incident stroke, and death after stroke incidence are displayed in Table 2. Among men, considerable disparities in all three transitions could be found. Belonging to the higher income group of >60% of the German pre-tax income reduced the risk of stroke incidence by 25%, the risk of death after stroke incidence by 18%, and the risk of death without incident stroke by 36%. In women, income disparities in all three transitions are less pronounced than in men. This holds especially for disparities in the risk of stroke incidence and death after stroke where only slight income differences between income groups could be observed (Table 2).

**Table 2:** Risks (HR) of stroke incidence, death without stroke, and death after stroke incidence of the higher income group compared to the lower income group by sex.

	Stroke incidence	Death without stroke	Death after stroke incidence
	HR (95%-CI)	HR (95%-CI)	HR (95%-CI)
**Men**			
Lower income (Ref.)	1	1	1
Higher income	0.75 (0.73–0.77)	0.64 (0.63–0.65)	0.82 (0.77–0.87)
**Women**			
Lower income (Ref.)	1	1	1
Higher income	0.96 (0.93–0.99)	0.88 (0.87–0.89)	0.94 (0.90–1.00)

HR Hazard Ratio; 95%-CI bootstrapped (with replacement) using 1000 replications; all analyses are controlled for age in single-year age groups (as second-degree polynomial) and for period

### Time trends in stroke incidence and mortality risks

With respect to changes between 2006–2008 and 2014–2016, the estimated proportional hazard models indicate a significant decrease in the risk of all three types of events appearing in both sexes as well as in both income groups. Among men and women, the risk of stroke incidence reduced to a similar degree in both income groups. These reductions amount to 7 to 10% over time. However, the risk of death after stroke incidence as well as without stroke decreased somewhat stronger among the higher income group. This tendency is more pronounced in men than in women (Table 3).

**Table 3:** Time trends in risks of stroke incidence, death without stroke, and death after stroke incidence by sex and income group (reference: period 1 (2006–2008)).

	Men				Women			
	Lower income		Higher income		Lower income		Higher income	
	HR	(95%-CI)	HR	(95%-CI)	HR	(95%-CI)	HR	(95%-CI)
**Stroke incidence**	0.93	(0.90–0.96)	0.91	(0.87–0.96)	0.91	(0.87–0.96)	0.90	(0.85–0.95)
**Death without stroke**	0.96	(0.94–0.98)	0.87	(0.85–0.90)	0.98	(0.96–0.99)	0.92	(0.89–0.95)
**Death after stroke incidence**	0.89	(0.83–0.95)	0.83	(0.75–0.90)	0.86	(0.82–0.90)	0.83	(0.75–0.91)

HR Hazard Ratio; 95%-CI bootstrapped (with replacement) using 1000 replications; all analyses are controlled for age in single-year age groups (as second-degree polynomial)

The age-specific hazard rates predicted by the proportional hazard incidence and mortality models were used to calculate the expected number of stroke-free life years and life years after stroke incidence. Overall, the predicted age-specific hazard rates fit the observed rates well (see supporting information S1 and S2 Figs).

### Trends in stroke-free and stroke-affected life years

Table 4 displays the expected number of remaining stroke-free and stroke-affected life years at age 50. In both time periods, men and women of the higher income group could expect more of their remaining years to be stroke-free life years than individuals of the lower income group. Between the two time periods, a significant increase in the number of life years spent without stroke of almost one year could be observed among men belonging to the higher income group (28.72 years in 2006–2008 to 29.61 years in 2014–2016). Among women and men of the lower income group no significant change in stroke-free life years emerged. The number of expected life years after stroke incidence in men increased significantly in both income groups. Whereas these increases indicate only a slight tendency to growing numbers of life years after stroke incidence among men belonging to the lower income group (0.95 years in 2006–2008 to 1.05 years in 2014–2016), the increase was more clearly in the higher income group (1.04 years in 2006–2008 to 1.25 years in 2014–2016). Among women, no significant changes in life years after stroke incidence could be found (Table 4).

**Table 4:** Life expectancy (life years) free of stroke, affected by stroke, and total life expectancy at age 50 by sex, period, and income group.

		Stroke-free		Stroke-affected		Total	
	Income	lower	higher	lower	higher	lower	higher
	Period						
**Men**	**2006–2008**	22.28	28.72*	0.95*	1.04*	23.23*	29.76*
		(22.06–22.51)	(28.48–28.98)	(0.91–0.99)	(0.99–1.10)	(22.99–23.46)	(29.50–30.02)
	**2014–2016**	22.66	29.61*	1.05*	1.25*	23.71*	30.86*
		(22.42–22.90)	(29.38–29.87)	(1.00–1.09)	(1.19–1.32)	(23.47–23.95)	(30.60–31.12)
**Women**	**2006–2008**	30.90	33.29	0.79	0.81	31.69	34.10
		(30.73–31.09)	(32.88–33.68)	(0.75–0.83)	(0.75–0.87)	(31.50–31.88)	(33.69–34.50)
	**2014–2016**	30.91	33.45	0.86	0.84	31.77	34.29
		(30.75–31.09)	(33.14–33.77)	(0.82–0.90)	(0.80–0.89)	(31.59–31.94)	(33.97–34.62)

95%-CI bootstrapped (with replacement) using 1000 replications

* significant difference in the number of life years between the two periods based on the reported 95%-CIs

## Discussion

Our study shows that income differences in stroke incidence and mortality are substantial, causing inequalities in stroke-free life years and the length of life affected by stroke. Moreover, time trends in these inequalities differ between sexes as well as between income groups. While men and women in the higher income group could expect more stroke-free life years than individuals in the lower income group, significant increases in stroke-free life years over time could only be found in men belonging to the higher income group. Overall, the number of life years affected by stroke is higher in men than among women. Whereas income differences in stroke-affected life years are pretty small in women, they are more pronounced in men. Due to the lower level of stroke mortality, a higher number of life years spent after stroke incidence can be reported among the higher income group. These differences in the number of stroke-affected life years in men increased over time. Among women no clear changes in lifetime with and without stroke could be found.

The general trend towards decreasing incidence rates and the gain of life years spent free of stroke can be assumed to be driven by improved prevention, which is the determining factor for morbidity compression. However, whether morbidity compression, morbidity expansion, or a dynamic equilibrium has taken place can only be decided by including information on mortality and by analysing developments of life years spent in morbidity. While incidence and mortality rates show considerable changes over time, lifetime affected by stroke remains quite stable. An exception to this has to be reported in men belonging to the higher income group where stronger changes in both, the number of life years with and without stroke could be found. In this group, decreases in incidence rates were counterbalanced by decreases in mortality rates, fostering extended periods of life spent after stroke incidence. However, stroke-free life years increased at a faster pace. Thus, the proportion of stroke-free life years on total life expectancy remained nearly unchanged, pointing towards a dynamic equilibrium in men belonging to the higher income group. While differences in absolute numbers of stroke-affected life years between men of the lower and the higher income group widened over time, the proportion of stroke-affected life years is comparable in both groups and barely changed between periods, indicating constant inequalities over time. As no significant changes in lifetime with and without stroke could be found, constant inequalities can also be assumed in women.

Our results are in line with studies reporting decreasing rates of incidence and mortality after stroke over time [3, 14, 15]. Studies investigating changes in risk factors in the German population found decreasing prevalence of hypertension, elevated cholesterol and glucose levels, physical inactivity, and smoking [36–38]. Previous research also suggests that widening disparities in the prevalence of stroke risk factors may foster differing trends in incidence and mortality [24]. With respect to changes in socioeconomic differences in health behaviour, widening inequalities in smoking among men [39] and physical inactivity in both sexes [40] have been reported. These changes in health behaviour may partly explain the stronger increase in stroke-free life years in the higher income group among men.

### Strengths and limitations

Our findings are based on large case numbers of stroke incidence and deaths without stroke and after stroke incidence, which permit to run separate survival models for all three types of events even when analyses are stratified for income groups. Furthermore, the detailed information on coded diagnoses and mortality allow to identify the time point of events precisely and to determine chronological order of events. As our data represent a complete insurance population, the analyses are unaffected by health-related nonresponse which could occur in surveys if individuals refrain from study participation for health reasons [41].

As health insurance data do not include any information on impairments or self-reported disabilities, trends in the consequences of stroke for daily living could not be taken into account. Furthermore, ICD-10 codes do not provide information on stroke severity. A study from Germany attempted to derive disease severity from health insurance claims data [42]. The authors proposed to include data on coma, artificial respiration, length of inpatient stay, physiotherapy and nursing care to specify stroke severity levels. However, in accordance with the authors’ conclusions, we refrained from including them as indicators are not validated sufficiently [42]. Previous studies show that low socioeconomic position is associated with higher stroke severity and poorer functional status after stroke [24, 25]. Thus, higher income may be associated with more life years affected by stroke but impairments might be less severe. Including information on disability could provide a deeper understanding of trends in impairments caused by stroke but would require a linkage of health insurance data and other data sources, e.g. survey data or data of official population statistics on care dependency.

The dataset do not provide information on causes of death. Thus, deaths after stroke were defined by the chronological order of events, which does not allow to distinguish trends on the basis of a clear differentiation between stroke-related and non-stroke-related mortality. However, the aim of the study was to investigate trends in stroke-affected life years which can be analysed based on overall mortality as the expected length of stay in the state of stroke is determined by the hazard of stroke incidence and of leaving the state due to death, irrespective of the underlying cause of death.

Due to differences in the social structure, total life expectancy of the insurance population is lower than that of the general population of Germany. While the distributions of sex and age are comparable, individuals having lower incomes and holding lower occupational positions are overrepresented in our data. However, this overrepresentation does not affect the results as all analyses are controlled for or stratified by income. Nevertheless, it cannot be completely ruled out that occupational distributions also differ within income groups from the general German population. However, as long as the distribution of occupations within income groups remains stable over time, the reported time trends in stroke-free and stroke-affected life years remain unaffected.

Among women, the differences between income groups in stroke incidence and mortality are less pronounced than among men. This might be due to using individual income instead of household income frequently used when analysing survey data. As our dataset does not allow for matching income information of spouses and no information on household composition is available, household income could not be used in this study. This may have led to an underestimation of financial resources, especially among women as the general income level is lower than in men [43]. Thus, individual income might be a stronger predictor for stroke incidence and mortality in men than in women. However, individual income has shown to be appropriate for studying health inequalities, though smaller gradients are observed compared to using household income [44].

## Conclusions

Our study shows substantial differences in stroke incidence and mortality between income groups, causing inequalities in the length of life spent free of stroke and lifespan affected by stroke. While no changes in stroke-affected life years have been found among women, lifetime spent after stroke increased in men, especially among the higher income group. However, irrespective of income the proportion of stroke-affected life years remain stable between the periods. Thus, income inequalities in men and women remain constant over time. Furthermore, constant proportions of stroke-free and stroke-affected lifetime point towards a dynamic equilibrium among men. Further research should investigate the developments in stroke severity and impairments due to stroke. Special emphasis should be given to the question whether these developments differ between socioeconomic groups, causing growing or narrowing inequalities in functional limitations and quality of life after stroke incidence.

## Supporting information

S1 Fig **Observed and predicted hazard rates of (a) stroke incidence, (b) death without stroke, and (c) death after stroke incidence of the low income group (≤60% of the German average income) for men and women by period. Data source: AOK Niedersachsen (statutory health insurance in Lower Saxony, Germany)** Predicted hazard rates are derived from proportional hazard multistate survival models with constant baseline hazards; all survival models are controlled for age in single-year age groups (as second-degree polynomial).
(PDF)

S2 Fig **Observed and predicted hazard rates of (a) stroke incidence, (b) death without stroke, and (c) death after stroke incidence of the higher income group (>60% of the German average income) for men and women by period. Data source: AOK Niedersachsen (statutory health insurance in Lower Saxony, Germany)** Predicted hazard rates are derived from proportional hazard multistate survival models with constant baseline hazards; all survival models are controlled for age in single-year age groups (as second-degree polynomial).
(PDF)
