# Supplementary material for: Income inequalities in stroke incidence and mortality: Trends in stroke-free and stroke-affected life years based on German health insurance data
Source: PLoS One. 2020 Jan 16;15(1):e0227541. doi: 10.1371/journal.pone.0227541 (PMC6964859; doi:10.1371/journal.pone.0227541)
Supplement: S1 Fig — Observed and predicted hazard rates of (a) stroke incidence, (b) death without stroke, and (c) death after stroke incidence of the low income group (≤60% of the German average income) for men and women by period. Data source: AOK Niedersachsen (statutory health insurance in Lower Saxony, Germany) Predicted hazard rates are derived from proportional hazard multistate survival models with constant baseline hazards; all survival models are controlled for age in single-year age groups (as second-degree polynomial). (PDF) [file pone.0227541.s001.pdf]

Supplementary material S1

PLOS One

Income inequalities in stroke incidence and mortality: trends in stroke-free and stroke-affected life years based on German health insurance data

Juliane Tetzlaff[1*], Siegfried Geyer[1], Fabian Tetzlaff[2¶], Jelena Epping[1¶]

[1] **Medical Sociology Unit, Hannover Medical School, Hanover, Germany**

[2] **Institute for General Practice, Hannover Medical School, Hanover, Germany**

S1 Fig: Observed and predicted hazard rates of (a) stroke incidence, (b) death without stroke, and (c) death after stroke incidence of the low income group (≤60% of the German average income) for men and women by period. Data source: AOK Niedersachsen (statutory health insurance in Lower Saxony, Germany)

a

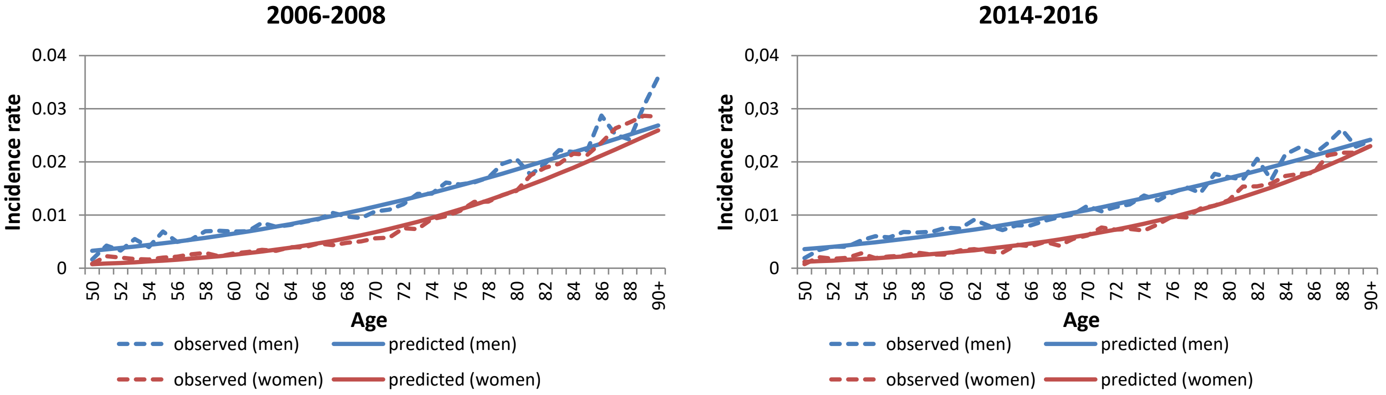

b

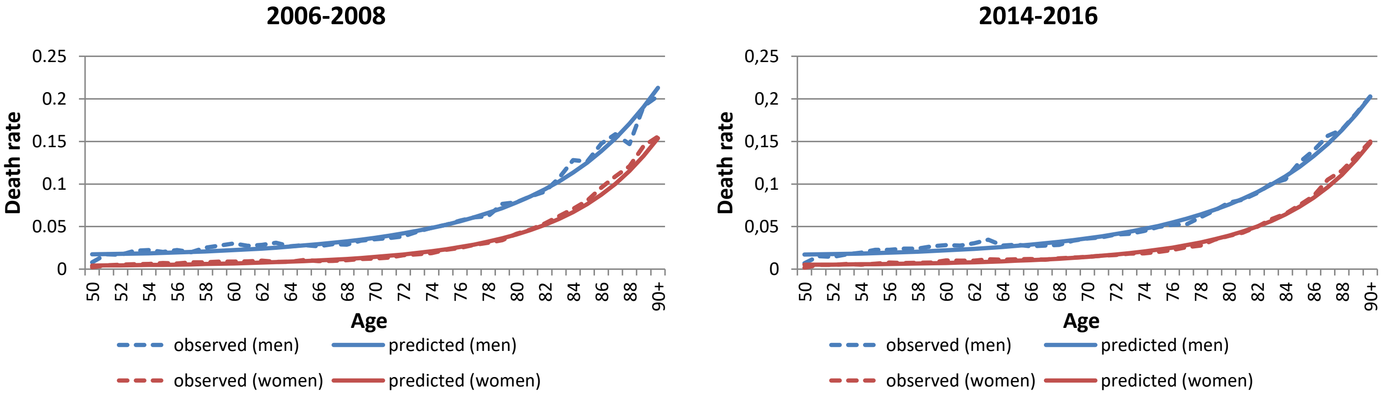

e

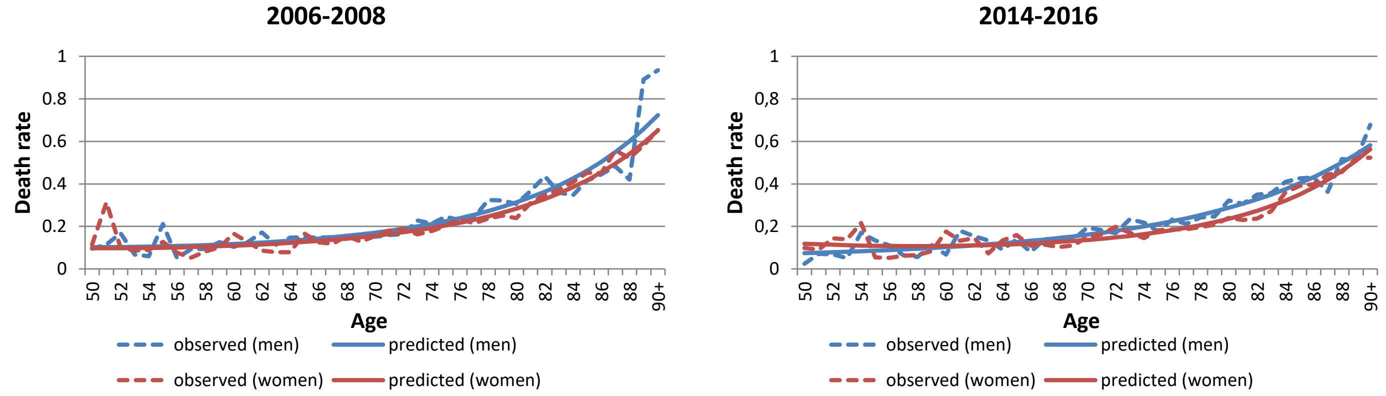

Predicted hazard rates are derived from proportional hazard multistate survival models with constant baseline hazards; all survival models are controlled for age in single-year age groups (as second-degree polynomial)
